# Comprehensive Approach to Phenotype *Varroa destructor* Reproduction in Honey Bee Drone Brood and Its Correlation with Decreased Mite Reproduction (DMR)

**DOI:** 10.3390/insects15060397

**Published:** 2024-05-29

**Authors:** Regis Lefebre, David Claeys Bouuaert, Emma Bossuyt, Lina De Smet, Marleen Brunain, Ellen Danneels, Dirk C. de Graaf

**Affiliations:** 1 Laboratory of Molecular Entomology and Bee Pathology (L-MEB), Department of Biochemistry and Microbiology, Ghent University, Krijgslaan 281, 9000 Ghent, Belgium; david.claeysbouuaert@ugent.be (D.C.B.); lina.desmet@ugent.be (L.D.S.); marleen.brunain@ugent.be (M.B.); dirk.degraaf@ugent.be (D.C.d.G.); 2 Honeybee Valley, Department of Biochemistry and Microbiology, Krijgslaan 281, 9000 Ghent, Belgium; embossuy.bossuyt@ugent.be (E.B.); ellen.danneels@ugent.be (E.D.)

**Keywords:** honey bee pathology, *Varroa destructor*, brood resistance, trait phenotyping

## Abstract

**Simple Summary:**

Since the mid-20th century, the parasitic *Varroa* mite has become a major threat for honey bee health worldwide. However, mature female mites are only able to reproduce in the honey bee colony’s brood cells, which are the hexagonal wax cells in which early bee larvae and pupae develop. By abundantly feeding on the larvae and pupae’s fat body tissues and hemolymph, the mites weaken the bees and act as an important vector for many honey bee viruses. Worldwide, distinct natural mite-surviving colonies have already been observed, which often show a range of *Varroa* resistance traits suppressing the mite’s population growth. One such trait is characterized by the suppression of mite reproduction in the brood cells of the developing bees. However, accounting for this trait in selection programs to improve resilience is hard, as the trait is labor- and time-intensive to evaluate. In this study, we propose an alternative, more holistic way of evaluating this *Varroa* resistance trait, reducing labor without impairing precision, which ultimately allows more colonies to be included in honey bee selection or breeding programs.

**Abstract:**

The mechanisms of action behind decreased mite reproduction (DMR) are still unknown, but current hypotheses state that DMR is the result of brood-intrinsic and/or external disturbances in the *V. destructor*—honey bee pupa signal interactions. For accurate and precise DMR phenotyping, sufficient single infested honey bee brood cells are required (e.g., 35), which requires extensive labor and time and may exclude many samples not reaching the threshold. We defined a new comprehensive trait called the ‘mean *V. destructor* reproduction rate’ (mVR), which describes the mean number of offspring mites per infested cell in the sample while compensating for the reduced number of offspring with increasing multiple infested cells. We found a significant correlation between mVR and DMR, allowing for an estimation of DMR based on the mVR only. When the mVR was calculated with 10 infested cells, we found an average variation in mVR of 16.8%. For the same variation in DMR determination, 40 single infested cells are required. This broader look at *V. destructor* resistance phenotyping can improve the applicability and effectiveness of traits related to *V. destructor* reproduction in honey bee breeding programs.

## 1. Introduction

Since its shift from the Eastern honey bee (*Apis cerana*) to the Western honey bee (*Apis mellifera*) during the early 20th century [1], the ectoparasitic mite *Varroa destructor* has become a major threat for apiculture. The mite feeds on the bee’s fat body tissues and hemolymph and affects its immune system, metabolism and winter survival chances [2,3,4,5]. In addition, *V. destructor* acts as an important vector for many honey bee viruses. For instance, a mutualistic symbiosis has been shown between the mite and deformed wing virus (DWV) as well as *V. destructor* infestation rates and DWV loads, both contributing to honey bee colony losses at a global scale [4,5,6,7,8,9,10]. Abiotic factors such as apiary colony density, climate, food availability and quality or insecticide exposure may intensify this negative host–parasite interaction [11,12,13,14,15,16,17,18,19].

Sustainable beekeeping aims for the selection and maintenance of locally adapted honey bees that can be managed without the need for treatment against pathogens. For example, natural mite-surviving colonies have already been established in Europe, Africa and America [20,21] and often possess a range of behavioral and individual *V. destructor* resistance traits frequently associated with lower mite population growth [21]. One of the earliest described defensive traits is suppressed mite reproduction (SMR) [22,23], which has been observed in several mite-surviving colonies, for instance, in Avignon (France), Gotland (Sweden) and Primorsky (Russia) [20,21,24,25]. SMR is a collective term for honey bee traits that affect mite reproductive success, such as delayed egg oviposition(s) by the foundress mite (fecundity-based), absence of male offspring and/or total absence of offspring (fertility-based) [26,27,28]. Von Virag et al., 2022, proposed the term decreased mite reproduction (DMR) instead of SMR, as the term ‘suppressed’ suggests that the trait is characterized by the complete absence of offspring only. Thus, DMR includes both fecundity-based reduction in mite reproduction (reduced mite reproduction, RMR) and fertility-based reduction in mite reproduction (mite non-reproduction or MNR) [28]. The exact underlying mechanisms of DMR are currently unknown, but since the reproductive cycle of *V. destructor* is highly dependent on host signals [29,30,31], it is now stated that DMR is the result of brood-intrinsic and/or external disturbances in this host–parasite signal interaction. This hypothesis is supported by the findings of Broeckx et al., 2019, in which eight single-nucleotide polymorphisms (SNPs) were found to be associated with drone brood resistance (DBR; MNR in drone brood) in a *V. destructor*-resistant population. They stated that some SNPs (found in different transporters) might cause lower brood pheromone production by the pupae, while another SNP in the dynein beta chain (a cytoskeletal motor protein involved in retrograde transport in insect olfactory neurons) could cause better sensing of these reduced pheromone concentrations by brood-caring worker bees [32]. DMR is also considered an indirect measure for *V. destructor*-sensitive hygiene (VSH), as colonies scoring high on VSH prefer to remove pupae with reproducing mites over pupae with non-reproducing mites, leading to lower proportions of reproducing mites in capped brood from VSH colonies [33,34,35]. However, the extent to which both traits are related is still under debate [27,36,37].

Both VSH and DMR are targeted in selection programs aiming for increased resilience to *V. destructor* by phenotyping participating colonies for the respective traits and breeding with only those queens with the best trait scores [38]. For example, to measure the extent of DMR in a colony, testers investigate capped brood under a microscope and determine the fraction of infested cells with non-reproducing mites and mites with fewer daughters than would be expected compared to the pupal age [26,28,39,40]. In such analyses, only single infested cells are used, as one cannot determine the reproductive successes of multiple foundresses in one cell, and the number of offspring per mother mite decreases with increasing numbers of mites per cell [41,42,43,44]. A reliable DMR estimation requires a minimum of 35 single infested cells, as recommended by several authors [26,27,39]. As the mean number of mother mites per infested cell decreases with decreasing infestation rates [42], reaching this minimum number of single infested cells requires a high number of inspected brood cells, which is labor-intensive and time-consuming [26,27]. Moreover, this threshold still holds an average variation in DMR estimations within the same sample of 20%, caused solely by which 35 single infested cells are being selected in the sample [26,27,39]. Furthermore, the repeatability of DMR measurements between worker brood samples of the same colony and sampling moment and inter-caste reproducibility between drone vs. worker brood has been shown to be low [28].

In this study, we investigated whether multiple infested cells could be incorporated in *V. destructor* reproduction phenotyping. After phenotyping drone brood samples for DMR as part of the Flemish Bee Breeding Program, only a limited number of brood samples contained sufficient single infested cells for accurate phenotyping (24 out of 514 samples). In this breeding program, sampling of drone brood between May and June is preferred over worker brood as drone brood is more attractive for *V. destructor* at low infestation levels in early spring and quick phenotyping is crucial for the communication of results and breeding purposes. To decrease the workload required for accurate phenotyping and increase the number of samples for which reliable results regarding mite reproduction could be gathered, we extended our screening by including multiple infested cells. The new comprehensive trait that emerged was called the ‘mean *V. destructor* reproduction rate’ (mVR) and describes the mean number of offspring per infested cell in the sample while compensating for the reduced number of offspring with increasing proportions of multiple infested cells. As the mVR can be considered an adapted version of DMR (including multiple infested cells), correlations between the two parameters were investigated. Overall, this report is a stepping stone to further develop robust and easy-to-implement protocols, improving the selection potential for traits related to *V. destructor* resistance in honey bees.

## 2. Materials and Methods

### 2.1. Colony Sampling and Data Collection

A total of 514 normal sized colonies (Simplex, Dadant Blatt, Langstroth or Zander format) with maximum one-year-old queens were sampled between 2018 and 2022 in the framework of the Flemish Bee Breeding Program (Supplementary Table S1). In this breeding program, one-year-old queens are tested in spring/summer for behavioral traits (such as gentleness and swarming) and resilience (e.g., virus and *Varroa* drone brood resistance). During summer, the results are communicated to the beekeepers, queen rankings are made and advice is given for further breeding. All sampled colonies were managed according to standard beekeeping practices. For each colony to be tested, the beekeeper was asked to provide at least 2 dm^2^ of 15–19-day-old capped drone brood (pink/purple eyed white pupae), sampled between May and June. The samples were frozen immediately after collection from the hive and kept in a cold chain (−20 °C) until further analysis. For each brood sample, capped cells were individually opened under a microscope and inspected for the presence of foundress mites and offspring mites (excluding eggs). In addition, the pupal ages of all opened cells were estimated according to [45]. The average pupal age of a brood sample was calculated as the average age of all collected pupae from that sample. Inspections were ceased if either 35 single infested cells were found or if 200 cells were opened. The infestation rate of a sample was calculated as the percentage of infested cells on the total number of opened cells.

### 2.2. Phenotyping Calculations

The threshold for calculating MNR, RMR and DMR was lowered to 20 single infested cells as only 24 samples were found with the recommended minimum of 35 single infested cells. To calculate MNR in a brood sample, the percentage of single infested cells without offspring over the total number of single infested cells was calculated. Mites that showed delayed onset of reproduction were considered reproductive for MNR calculations. To calculate RMR, the percentage of single infested cells containing mites with delayed oviposition from the total number of single infested cells was calculated. Delayed oviposition was determined according to the descriptions of Martin, 1997 [46]. For each sample, DMR was determined by the sum of percentages of MNR and RMR for that sample.

To determine the mean number of offspring mites per mother mite (O/M), the total number of offspring mites in the sample was divided by the total number of mother mites in that sample. The mean number of mother mites per infested cell (M/I) was calculated by dividing the total number of mother mites by the total number of infested cells in a sample. As O/M is highly correlated with M/I due to the negative effect of multiple infestation on *V. destructor* fecundity, the ‘mean *V. destructor* reproduction rate’ (*mVR*) for a brood sample was calculated by multiplying the mean number of offspring mites per mother mite (O/M) with the mean number of mother mites per infested cell (M/I) and the absolute value of the slope of the linear relationship between O/M and M/I (=*m*):

$$mVR=\frac{total\;number\;of\;offspring\;mites}{total\;number\;of\;mother\;mites}\times \frac{total\;number\;of\;mother\;mites}{total\;number\;of\;infested\;cells}\times m$$

### 2.3. Statistics

R version 3.6.1 was used for data analysis, visualization and resampling iterations. Correlation analyses were conducted by either Pearson or Spearman correlation, depending on whether the data were normally distributed or not. All tests were checked for and complied with the required assumptions.

## 3. Results

### 3.1. Data Overview and Filtering

Of the 514 examined drone brood samples (one per colony), no infested cells were found in 107 samples (21%). In total, 74,613 cells were opened, of which 3951 were infested with one adult mite and 1772 cells with multiple mother mites. On average, 11 infested cells were found per sample. Based on the increase in variability of the mean number of offspring mites per mother mite (O/M) with the decreasing number of infested brood cells per sample, a minimum of 10 infested cells was set for further analysis (Supplementary Figure S1). Brood samples with an average pupal age younger than 16.5 days or older than 18.5 days were excluded for further analyses as foundress mites could not express full fecundity in brood younger than 16.5 days and older daughter mites were difficult to distinguish from mother mites when the brood was older than 18.5 days. This is reflected in the decrease in O/M in samples with an average pupal age younger than 16.5 days (Spearman; r = 0.36, *p* = 0.002) and samples with an average pupal age greater than 18.5 days (Spearman; r = −0.56, *p* = 1.49 × 10^−5^) (Supplementary Figure S2). The average age did not significantly influence O/M after selecting for average pupal age per sample between 16.5 and 18.5 days (Spearman; r = −0.16, *p* = 0.123). After filtering by average pupal age and a minimum of 10 infested brood cells, 88 samples remained for further analyses. In these samples, the portions of infested cells with multiple mother mites (%MIC) varied from 0 up to 63%.

Of the remaining 88 samples, 35 had at least 20 single infested cells (lowered threshold) and gave the minimal necessary information to calculate MNR, RMR and DMR. The infestation rates of these 35 samples ranged between 6 and 63.5% and %MIC varied from 0 to 50%.

### 3.2. Mean V. destructor Reproduction Rate

**Figure 1:**
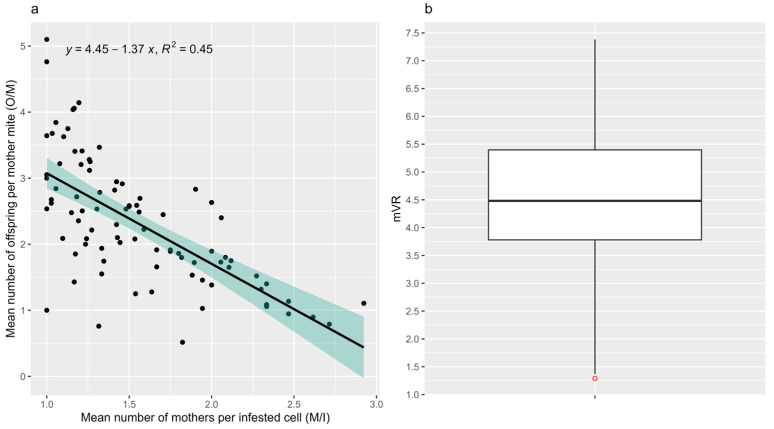
Correlation and linear regression between O/M and M/I (**a**) and normal distribution of mVR (**b**). The mean number of offspring mites per mother mite (O/M) is significantly correlated with the mean number of mother mites per infested cell (M/I) (Spearman; r = −0.69, *p* = 9.48 × 10^−14^, N = 88). The mVR is calculated as O/M × M/I × 1.37 and is normally distributed in the 88 samples (Shapiro; *p* = 0.607).

The mean number of offspring mites per mother mite (O/M) was significantly correlated with the mean number of mother mites per infested cell (M/I) (Spearman; r = −0.69, *p* = 9.48 × 10^−14^, N = 88), with the slope of the linear regression curve being −1.37 (Figure 1a). As such, mVR was calculated for each of the 88 samples by multiplying O/M with M/I and the absolute value of the slope (mVR = O/M × M/I × 1.37). Figure 1b shows a normal distribution of mVR over all 88 brood samples (Shapiro; *p* = 0.607), with a minimum and maximum mVR of 1.29 (outlier) and 7.38, respectively, and a mean of 4.52. The mean *V. destructor* reproduction rate was uncorrelated with M/I (Spearman; r = −0.11, *p* = 0.321) and %MIC (Spearman; r = −0.01, *p* = 0.900). Moreover, the observed mVRs were not significantly correlated with the total number of infested cells in the samples (Spearman; r = 0.21, *p* = 0.051), the total number of mother mites in the samples (Spearman; r = 0.18, *p* = 0.090) or the infestation level in the samples (Spearman; r = 0.09, *p* = 0.402). 

**Figure 2:**
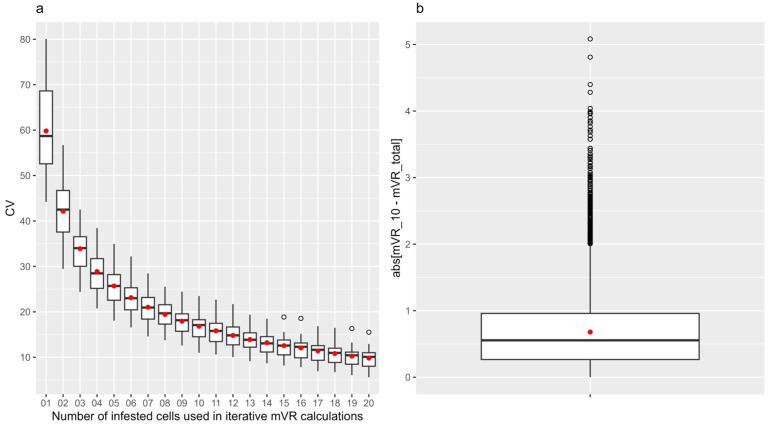
Iterative sampling for (**a**) determination of the average coefficient of variation of mVR for different numbers of infested cells and (**b**) distribution of the absolute difference between mVR calculated with 10 infested cells vs. total number of infested cells. For each of the 24 samples, the coefficient of variation (CV) was determined for different numbers of infested cells (*i* = 1 to 20). The CV in an iteration series (with 1000 random samplings) was calculated by dividing the standard deviation in the iteration series by the average mVR for that iteration, multiplied by 100. (**a**) shows the distributions in CVs across all 24 samples in function of the number of infested cells used for mVR calculations (*i*). In each of the 24 samples, the 1000 absolute differences between the mVR calculated with 1000 times 10 random infested cells and the total number of infested cells (N ≥ 30) were calculated. (**b**) shows the distribution of all 24,000 absolute differences. Red dots represent means.

For each of the 24 samples with at least 30 infested cells, 1000 random resampling iterations were performed to calculate the coefficient of variation (CV) in mVR for each number of analyzed infested cells, i, varying from one to twenty (Figure 2a). In a single sampling, i random infested cells were selected in the sample and the mVR was calculated. This was repeated 1000 times per iteration series, and the CV was calculated by dividing the standard deviation of the 1000 obtained mVRs with the mean mVR and multiplying this factor by 100. Thus, we acquired 20 CV values per brood sample, as i ranged from one to twenty. Per value of i, we obtained 24 CV values (24 samples). Calculating the mVR with 10 infested cells resulted in, on average, a coefficient of variation of 16.8% (Figure 2a). Moreover, in these 24 samples, the average and median difference in iteratively calculated mVRs with 10 random infested cells versus the maximum number of infested cells were 0.68 and 0.55, respectively (N = 24,000; Figure 2b). Interestingly, only 597 (2.5%) of the 24,000 iterative calculations resulted in an absolute difference higher than 2 (i.e., outliers).

### 3.3. Correlation with DMR

**Figure 3:**
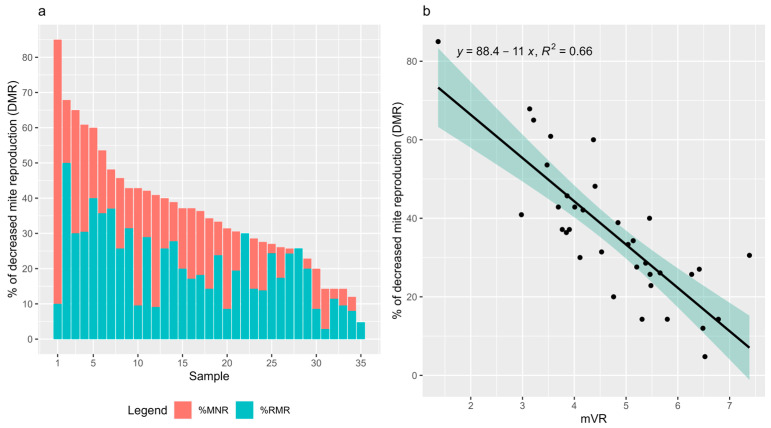
Distribution of MNR, RMR and DMR (**a**) and correlation of mVR with DMR (**b**) in the 35 samples with at least 20 single infested cells. MNR was calculated as the percentage of single infested cells without offspring over the total number of single infested cells in the sample. Mites that showed delayed onset of reproduction were considered reproductive for MNR calculations. RMR was calculated as the percentage of single infested cells containing mites with delayed oviposition over the total number of single infested cells. The percentage of DMR was determined by the sum of percentages of MNR and RMR. N = 35.

In the 35 samples for which MNR, RMR and DMR could be calculated, the percentage of DMR varied between 85% and 4.8% (Figure 3a), with an average of 36.2%. In these samples, the average MNR was 15.4% and the average RMR was 20.8%. Moreover, the average proportions of MNR and RMR in DMR were 38.9% and 61.1%, respectively.

In these 35 samples, mVR was significantly correlated with the percentage of DMR (Pearson: r = −0.81, *p* = 2.88 × 10^−9^, N = 35) (Figure 3b). Moreover, DMR was uncorrelated with the total number of infested cells in the sample (Spearman, r = −0.06, *p* = 0.729), the total number of mother mites in the sample (Spearman; r = −0.06, *p* = 0.734) and the infestation level in the sample (Spearman; r = −0.10, *p* = 0.553).

## 4. Discussion

Despite the large number of cells opened, only a limited number of brood samples had the required minimal number of single infested cells (in this study set at 20), and only 24 samples (4.7%) attained the prescribed 35 single infested cells to calculate MNR, RMR and DMR [26,27,39]. Other studies mention having between 43 and 72% of samples with more than 35 single infested cells for worker brood [26,28] and 87% for drone brood [28], although this number highly depends on the number of cells opened and the infestation levels of the samples. The presented results are in line with the reported difficulties of the low proportion of single infested cells in brood samples with either low or high infestation rates and the high workload to phenotype samples for MNR, RMR and DMR [26,27,28,47]. More specifically, highly infested samples will contain a high number of multiple infested cells and thus low numbers of single infested cells for accurate calculation of MNR, RMR and DMR [42]. Low infestation rates will result in low numbers of (single) infested cells among the opened cells, thus making the process time-consuming too. Therefore, obtaining a parameter for *Varroa* reproduction in brood requiring fewer (single) infested cells but retaining sufficient accuracy and precision could lower the workload and time needed for phenotyping, especially in the framework of bee breeding programs. In this study, the average and median difference in mVR between 1000 times 10 random selected infested cells and the maximum number of infested cells (for 24 samples with at least 30 infested cells) were 0.68 and 0.55, respectively. This sufficiently low average and median difference suggest that 10 infested cells are adequate to differentiate between high and low mVR values, especially when applied in, for example, negative selection programs [48]. Through a resampling analysis and determination of the average coefficient of variation in mVR across 24 samples with N infested cells (N ≥ 30), we demonstrated again that 10 infested cells is an acceptable minimum for mVR calculations, as the average CV for 10 infested cells across the 24 samples was only 16.8%. In other words, when the mVR is calculated with 10 infested cells, the variation in mVR caused by which 10 infested cells are selected in the sample will be, on average, 16.8%. For the same variation in DMR determination, 40 single infested cells are required [27]. If less variation is desired, more infested cells should be used for mVR calculation. For example, if 20 infested cells are used for mVR calculation, the expected average variation will be 9.8%. This latter average variation in DMR calculations can only be obtained when at least 59 single infested cells are analyzed [27]. A recent study showed that although measuring the suppression of mite reproduction with only 10 single infested cells is comparatively inaccurate and has low repeatability, selecting for the trait is still effective due to the high heritability value of the trait [49]. However, in the framework of selection programs working with accurate queen ranking, such as the Flemish Bee Breeding Program, an expected average variation in DMR of 42% (when using only 10 single infested cells) will have significant effect on the final ranking and results communication.

In the current study, the mVR allowed for the phenotyping of 2.5 times as many samples (88 compared to 35 for DMR (with lowered threshold)). An additional advantage of the mVR is that data can be gathered as the total number of infested cells (single and multiple infested), the total number of adults and the total number of offspring mites, facilitating a faster and more comprehensive phenotyping procedure compared to noting down information for each cell individually. Factors underlying the mVR such as MNR, RMR, VSH or the interaction between different traits [34] all impact the resulting reproduction rate. This is especially reflected in the significant correlation between mVR and DMR found in this study, which allows for an estimation of the DMR value based on the mVR value of that colony. The average DMR score in the 35 samples with at least 20 single infested cells was 36.2%, which is comparable to the average DMR of 32.8% reported in a recent pan-European study [26]. In the latter study, 15.9% of the sampled colonies showed a percentage DMR equal to or greater than 50%. In our study, this was the case for 6 of the 35 samples (17.1%). Moreover, 51.4% of the colonies in our study showed a DMR score between 20 and 40%, which is comparable to the results in the pan-European study (57.5%). However, we should note that the two studies differ in the total number of samples (35 vs. 414 samples in the pan-European study), the minimal number of single infested cells required for calculations (20 vs. 10 in the pan-European study) and the brood type (drone vs. worker brood). Moreover, contrary to other studies, no distinction was made between female and male mite offspring in the current study, with a probable overestimation of the mite reproduction as foundress mites producing only male offspring were counted as reproductive instead of non-reproductive. On the other hand, for DMR calculation, these foundress mites will be classified as showing delayed oviposition and will also lower the mVR value due to the low number of offspring mites.

It should be noted that the current correlation between mVR and DMR is based on 35 samples, with a lowered threshold for DMR calculations (20 single infested cells instead of 35). Including more samples with sufficient single infested cells (e.g., 35 or more) may reinforce this correlation. Further research on a bigger dataset may be necessary to confirm the linear correlation with extreme values (DMR > 70% and <10%) and to assess the repeatability of the mVR in samples taken from different brood frames of the same colony, over different sampling moments and its relevance in worker brood. These latter proposed analyses are especially important to conduct, as in this study, each participating beekeeper provided only a limited number of samples per year, resulting in low repetition of each environmental condition. The applicability of the mVR for worker brood samples could not be evaluated in the current study as worker brood samples are not being collected by beekeepers participating in the Flemish Bee Breeding Program due to the higher impact of worker brood sampling on overall colony development.

Moreover, we should note that the value of ‘m’ (i.e., 1.37) is still specific for the dataset used here, and that it is yet unknown whether the value is generalizable or not. A follow-up study should investigate whether the value of ‘m’ differs between different datasets, constructed from either different sampling moments, other sampled colonies, various sampled subspecies and/or distinct geographical locations. If the value of ‘m’ is not generalizable, one should always determine it by plotting the mean number of offspring mites per mother mite (O/M) in function of the mean number of mother mites per infested cell (M/I) in the newly obtained dataset. Comparing exact mVR values between different datasets should then be performed carefully, as the exact mVR value is influenced by the factor ‘m’. On the other hand, intra-dataset comparison remains possible. Simply removing ‘m’ from the formula would not influence any correlations between the mVRs and other factors such as DMR in the study reported here, as all mVR calculations included the multiplication with 1.37. However, we prefer to retain it in the formula as this factor mirrors the actual average decrease in mean number of offspring mites per increase in, on average, one mother mite per infested cell (=meaning of the slope of the regression curve). Moreover, the reflection of this factor is especially useful to discriminate subtle differences in extreme cases (with high biological meaning), which can be found in either the first or fourth quartile of the mVR distribution. These two quartiles are also the most important in selective breeding programs, either in positive (selection of colonies with low mVR and thus high DMR) or negative (exclusion of high mVR and thus low DMR) selection programs, respectively.

In general, the mVR can be considered an adjusted protocol for measuring DMR. The reason for defining it as a new trait instead of a new protocol is because the relationship between DMR in single infested cells and DMR in multiple infested cells is currently unknown. Thus, further research is needed to investigate to what extent brood resistance traits differ in single and multiple infested cells.

## 5. Conclusions

Most of the traits used in breeding programs across Europe revolve around lowering the reproduction rate of the *V. destructor* mite [50]. The use of the mVR as a breeding tool encompasses the effectiveness of the sum of all brood-related resistance traits, thereby selecting for the most suitable genetic background and not for one specific trait, while reducing the phenotyping workload and increasing the number of samples for which sufficient data can be gathered. Altogether, this broader look at resistance against *V. destructor* mites can improve the applicability and effectiveness when incorporating traits related to *V. destructor* reproduction in breeding programs worldwide.

## Data Availability

The datasets used and/or analyzed during the current study are available from the corresponding author on reasonable request.

## Supplementary Materials

The following supporting information can be downloaded at: https://www.mdpi.com/article/10.3390/insects15060397/s1, Figure S1: Mean number of offspring mites per mother mite (O/M) in function of number of infested brood cells for all samples with at least 1 infested cell; Figure S2: Mean number of offspring per mother mite (O/M) in function of average pupal age; Table S1: Number of drone brood samples per beekeeper and year.
