# Supplementary material for: Comprehensive Approach to Phenotype Varroa destructor Reproduction in Honey Bee Drone Brood and Its Correlation with Decreased Mite Reproduction (DMR)
Source: Insects. 2024 May 29;15(6):397. doi: 10.3390/insects15060397 (PMC11203922; doi:10.3390/insects15060397)
Supplement: Supplementary file 1 [file insects-15-00397-s001.zip › Supplementary Figures SI Insects.pdf]

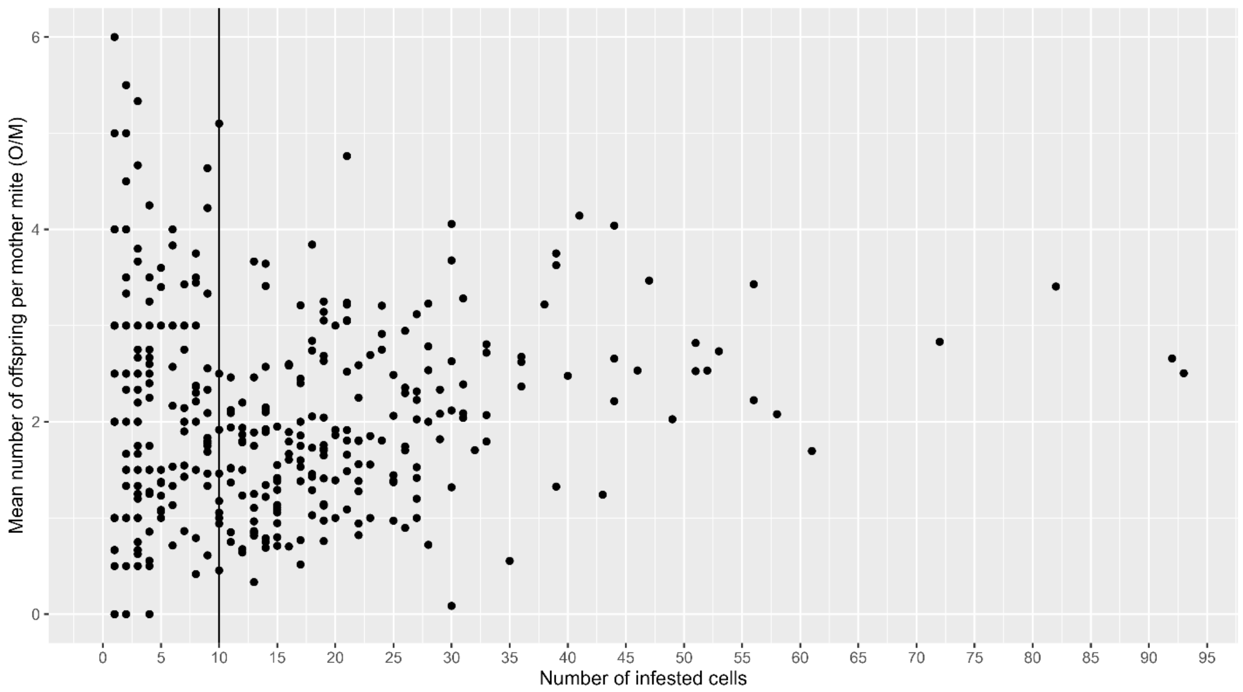

Supplementary Figure S1: Mean number of offspring per mother mite (O/M) in function of number of infested brood cells for all samples with at least 1 infested cell. O/M is calculated as the total number of offspring mites (males, protonymphs and deutonymphs) in a sample divided by the total number of mother mites in that sample. Based on the increase in variability of O/M with decreasing number of infested brood cells per sample, a minimum of 10 infested cells was set for further analyses.

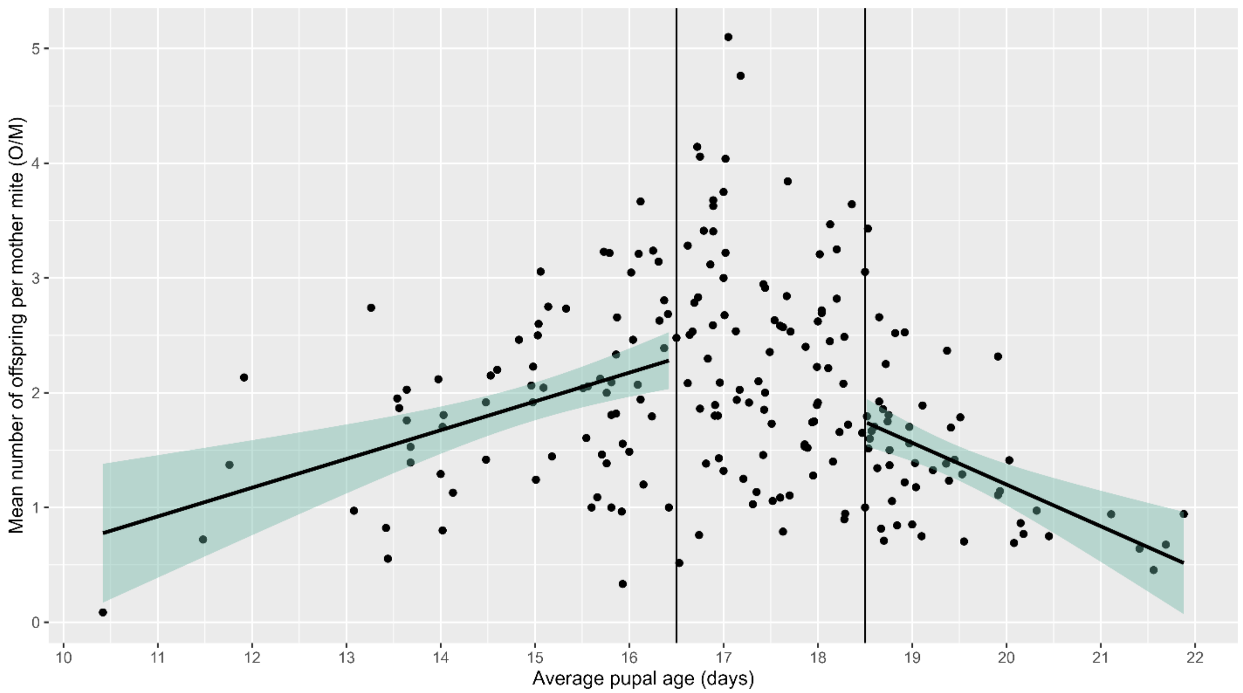

Supplementary Figure S2: Mean number of offspring per mother mite (O/M) in function of average pupal age. Figure S2 shows the relation between the average age of the drone pupae (in days) and the mean number of offspring per mother mite (O/M) in all samples with at least 10 infested cells. Foundress mites could not express full fecundity in brood younger than 16.5 days and older daughter mites were difficult to distinguish from foundress mites when the brood was older than 18.5 days. This is reflected in the decrease in O/M in samples with an average pupal age younger than 16.5 days (Spearman; r= 0.36, p = 0.002) and samples with an average pupal age greater than 18.5 days (Spearman; $r = -0.56$, $p = 1.492*10^{-5}$).
