# Supplementary material for: Comprehensive Approach to Phenotype Varroa destructor Reproduction in Honey Bee Drone Brood and Its Correlation with Decreased Mite Reproduction (DMR)
Source: Insects. 2024 May 29;15(6):397. doi: 10.3390/insects15060397 (PMC11203922; doi:10.3390/insects15060397)
Supplement: Supplementary file 1 [file insects-15-00397-s001.zip › Supplementary Table S1.pdf]

Supplementary Table S1. Number of drone brood samples per beekeeper and year. Beekeepers are anonymized with numbers.

Beekeeper	Year					**Total**
	2018	2019	2020	2021	2022	
1		2		3	6	**11**
2					3	**3**
3					1	**1**
4	3	6	6	2		**17**
5		8	4	6	4	**22**
6			5	5		**10**
7		6				**6**
8				6		**6**
9					1	**1**
10				3		**3**
11				1		**1**
12			2	5	2	**9**
13	2		12	12	12	**38**
14			5			**5**
15			2			**2**
16			2			**2**
17		1				**1**
18					1	**1**
19			1	8		**9**
20				3	3	**6**
21					1	**1**
22			6	6	8	**20**
23			2	4	4	**10**
24					1	**1**
25			5	4		**9**
26				6		**6**
27				1	4	**5**
28			3			**3**
29				1		**1**
30				2		**2**
31				2		**2**
32		6		7		**13**
33					5	**5**
34					2	**2**
35					2	**2**
36			11	12		**23**
37		9	7	4		**20**
38				1	1	**2**
39	1		1	4		**6**
40			3	9	1	**13**
41				1		**1**

**Supplementary Table S1.** Continued						
42		3		3	2	**8**
43				3	4	**7**
44			2			**2**
45		1	1	6		**8**
46				4		**4**
47			5	8		**13**
48				4	3	**7**
49				2		**2**
50		6	6	6	8	**26**
51			6	6	5	**17**
52		3	3	7	5	**18**
53					3	**3**
54			2			**2**
55			1		2	**3**
56			4			**4**
57				3	7	**10**
58		8	7	12	10	**37**
59				5		**5**
60			1			**1**
61			3	4	5	**12**
62				3		**3**
63		5	5	4	7	**21**
**Total**	**6**	**64**	**123**	**198**	**123**	**514**
